# Using routine clinical and administrative data to produce a dataset of attendances at Emergency Departments following self-harm

**DOI:** 10.1186/s12873-015-0041-6

**Published:** 2015-07-16

**Authors:** C. Polling, A. Tulloch, S. Banerjee, S. Cross, R. Dutta, D.M. Wood, P.I. Dargan, M. Hotopf

**Affiliations:** King’s College London, Academic Department Psychological Medicine, Institute of Psychiatry, 10 Cutcombe Road, London, SE5 9RJ UK; Clinical Toxicology, Guy’s and St Thomas’ NHS Foundation Trust and King’s Health Partners, London, UK; King’s College London, London, UK; Health Services and Population Research Department, King’s College London, Institute of Psychiatry, De Crespigny Park, London, SE5 8AF UK

**Keywords:** Self-harm, Emergency departments, Electronic health records, Hospital episode statistics

## Abstract

**Background:**

Self-harm is a significant public health concern in the UK. This is reflected in the recent addition to the English Public Health Outcomes Framework of rates of attendance at Emergency Departments (EDs) following self-harm. However there is currently no source of data to measure this outcome. Routinely available data for inpatient admissions following self-harm miss the majority of cases presenting to services.

We aimed to investigate (i) if a dataset of ED presentations could be produced using a combination of routinely collected clinical and administrative data and (ii) to validate this dataset against another one produced using methods similar to those used in previous studies.

**Methods:**

Using the Clinical Record Interactive Search system, the electronic health records (EHRs) used in four EDs were linked to Hospital Episode Statistics to create a dataset of attendances following self-harm. This dataset was compared with an audit dataset of ED attendances created by manual searching of ED records. The proportion of total cases detected by each dataset was compared.

**Results:**

There were 1932 attendances detected by the EHR dataset and 1906 by the audit. The EHR and audit datasets detected 77 % and 76 % of all attendances respectively and both detected 82 % of individual patients. There were no differences in terms of age, sex, ethnicity or marital status between those detected and those missed using the EHR method. Both datasets revealed more than double the number of self-harm incidents than could be identified from inpatient admission records.

**Conclusions:**

It was possible to use routinely collected EHR data to create a dataset of attendances at EDs following self-harm. The dataset detected the same proportion of attendances and individuals as the audit dataset, proved more comprehensive than the use of inpatient admission records, and did not show a systematic bias in those cases it missed.

## Background

Self-harm is a significant public health issue in the UK [1]. It is strongly associated with the presence of mental disorders [2] and is the single strongest risk factor for future suicide [3]. At a population level, treatment of the consequences of self-harm places a significant burden on health services.

Population level assessments of the incidence of self-harm in England are most commonly based on Hospital Episode Statistics (HES). HES are administrative data on all admissions, outpatient appointments and Emergency Department (ED) attendances at National Health Service (NHS) hospitals in England. Rates of admission related to self-harm are used as the indicator to represent mental health and well-being in Public Health England’s Area Health Profiles [4], and have been used to study geographical variations in self-harm [5, 6].

However, HES admission statistics have limitations. They can only represent the proportion of self-harm that results in a hospital admission, and so miss presentations with self-harm that are seen and discharged from EDs without requiring admission [4]. Hawton et al. [1] made an estimate of approximately 220,000 self-harm presentations to EDs in England annually, by extrapolating from data collected at three centres by the Multi-Centre Study of Self-Harm in 2000 & 2001. In contrast HES admissions data for 2000/01 contains 68,090 admissions with self-harm coded as the cause [7]. This suggests that the majority of self-harm receiving emergency medical attention is not included in HES inpatient data. Additionally, criteria for admission following self-harm varies between hospitals, potentially introducing bias to comparisons in rates of self-harm between hospitals and areas.

In recognition of these limitations, the recent update of the Public Health Outcomes Framework now includes ED attendances with self-harm as a key indicator [8]. HES data are collected for ED attendances, however comparison to the official source used for ED attendance figures, the Quarterly Monitoring of Accident and Emergency (QMAE) shows that a significant proportion of ED attendances are missing from this dataset. Furthermore completion of data regarding reason for presentation is low, limiting its value as a source of routine data on presentations for self-harm [9]. The Public Health Outcomes Framework acknowledges that, at present, data for the indicator can only be estimated from monitoring data from the Multicentre Study on Self-harm in the three cities the study covers, describing it as a “data source that needs further development” [8].

Where research has been carried out on ED presentations for self-harm it has used data sets assembled by searching ED notes and/or getting ED and psychiatric staff to complete audit forms [1, 10]. These procedures require ongoing research worker time and co-operation from clinical workers in the hospital involved and so are considerably more labour intensive and expensive than using routine clinical data.

We aimed i) to test whether a dataset of ED presentations could be produced using a combination of routinely collected data from electronic health records and HES and ii) to validate the dataset against another produced using manual searches of ED notes and audit forms by (a) comparing the proportion of cases detected and (b) checking for systematic differences between cases detected and cases missed.

## Methods

### Location

South London and Maudsley NHS Foundation Trust (SLaM) provides all the NHS secondary mental health services for the London boroughs of Lambeth, Southwark, Lewisham and Croydon, serving a population of 1.2 million people. The four boroughs are served by four EDs; St Thomas’ Hospital, King’s College Hospital, Croydon University Hospital and University Hospital Lewisham. SLaM provides 24 h Psychiatric Liaison services in all four EDs, staffed by psychiatric liaison nurses and psychiatrists. All four EDs have policies of referring all attendees with self-harm for a psychiatric assessment and of recording these referrals regardless of whether individuals wait to be seen.

### Definition of self-harm

We used the National Institute for Health and Care Excellence (NICE) definition of self-harm; “any act of self-poisoning or self-injury carried out by an individual regardless of motivation” [11]. Presentations were excluded if the individual had previously presented to an ED with the same episode of self-harm or if the episode had occurred more than seven days ago. Any ingestion of non-recreational drugs above the prescribed dose identified as self-harm by the individual or ED staff was coded as self-poisoning. Use of recreational drugs was coded as self-poisoning where the patient reported intent to self-harm. Episodes were coded as self-injury where any intentionally self-inflicted injury, however superficial, had occurred but not where threats or gestures to self-harm had not resulted in injury. All attempted hanging, jumping from a height and immersion in water with intent to drown was coded as self-harm and categorised as “other” regardless of whether injuries were sustained.

### Data sources

#### Clinical Records Interactive Search

Since 2006 all patient records within SLaM have been stored in an electronic health record (EHR). Any contact an individual has with mental health services will create a record within the EHR. Every entry must be placed under a specific team and coded from a choice of locations which includes Emergency Departments. The Clinical Record Interactive Search (CRIS) system [12], supported by the NIHR Specialist Biomedical Research Centre for Mental Health at SLaM and the Institute of Psychiatry, King’s College London, was developed in 2008 to enable researchers to search and retrieve anonymised clinical records. CRIS contains all the data stored within SLaM’s electronic clinical records with personal identifiers removed. As of 23^rd^ April 2014 it contains clinical records on 231,688 patients, 99,026 of whom had contact with SLaM between 1^st^ April 2009 & 31^st^ December 2011, the period of interest for this study.

Work within CRIS is covered by a database approval from Oxfordshire REC C granted in September 2008 (08/H0606/71 + 5). CRIS has a rigorous security model, full details of which are published elsewhere [12]. The use of CRIS for this project was approved by the CRIS Oversight Committee which is chaired by a mental health service user and reviews all applications to use the Case Register. Patients with records in the EHR can ask to have their data removed from CRIS if they do not wish for it to be used for research, although, at the time of this research, only two individuals had done so.

#### Hospital Episode Statistics data

CRIS has been linked with HES data for both Admitted Patient Care and ED episodes of care. Static extracts of HES data are linked to CRIS data within the Health and Social Care Information Centre and provided to the Biomedical Research Centre for Mental Health with all identifiers removed. HES data is available within CRIS for all patients who have had any contact with SLaM services since 2006, regardless of where they were living at the time of their hospital use, and additionally for all people resident within the four boroughs SLaM serves at the time of their hospital use. Linked HES data were available up to the end of 2011, with ED data available from 2009/10 financial year onwards. Information on data completeness for HES ED data is available for 2010/11. For the 2010/11 year HES ED data were missing 8.8 % of attendances in the four hospitals [13].

#### SHIELD

SHIELD is a three year service improvement project that was funded at the time of this work by the Guy’s and St Thomas’ Charitable Trust. It collects data on attendances to King’s College Hospital and St Thomas’ Hospital EDs by individuals who have self-harmed. Two database scientists search the discharge and/or presentation diagnosis entered in the ED electronic record using a list of keywords relating directly to possible self-harm attendances and/or recreational drug use (e.g., overdose, self-poisoning, self-harm, drug overdose). In addition, common clinical conditions that could be associated with the acute toxicity of drugs or other compounds were also identified (e.g., out of hospital cardiac arrest, seizures). These keywords were developed through an iterative process of audit and include general terms found to be used as the diagnosis in cases of self-harm and acute recreational drug toxicity. The ED and/or inpatient hospital notes for cases identified by this search process are reviewed and coded according to whether the attendance was for self-harm, acute recreational drug toxicity or other types of poisoning (e.g., accidental/unintentional). In addition, the psychiatric liaison teams in both EDs are asked to complete a data form summarising key components of the psychosocial self-harm assessment after assessing anyone presenting with self-harm. The database scientists review the written hospital (ED and if necessary inpatient) records of all of these cases and enter data on the cases on to the database.

SHIELD data collection is approved under the clinical governance procedures by the Caldicott Guardians of Guy’s and St Thomas’, King’s College Hospital and South London and Maudsley NHS Trusts. The linkage of SHIELD and CRIS data for the validation process was approved by all three Caldicott Guardians.

### Developing the EHR dataset

#### Case identification

In the EHR dataset, an ED attendance by any given individual was defined by both of two criteria being met:

1. the presence of a HES ED attendance or, alternatively, a CRIS record of a period of treatment by an ED psychiatric liaison team.
2. the presence of a structured assessment form, free text note or correspondence item in CRIS with an entry date within twelve hours of (1), which was either entered by an ED liaison team or was recorded as having physically occurred in the ED.

Records from 1^st^ April 2009 to 31^st^ December 2011 were retrieved. The contents of the items described in (2) above were flagged if they contained any of a list of keywords related to self-harm, suicide attempts and suicidality. The flagged entries were read by one of two coders, CP and SB, and coded for presence of self-harm, type of self-harm and whether alcohol had been consumed at the time of self-harm or in the preceding 6 h. A sample of the first 200 individuals by numerical study ID, representing 346 ED attendances and 436 clinical record entries was coded by both coders and tested for inter-rater reliability of the coding of presence and type of self-harm using a kappa statistic.

Separately, HES inpatient data were used to identify individuals with an ICD-10 code for self-harm (X60-X84) who were admitted through EDs. Those attendances that did not already appear in the dataset created from ED records were identified.

### Validation of dataset

Data for 2011 from two of the four EDs, St Thomas’ Hospital and King’s College Hospital, were available from both the SHIELD dataset and the EHR dataset. These datasets were compared for level of agreement. EHR and SHIELD records were linked by staff within the Clinical Data Linkage Service at the Biomedical Research Centre for Mental Health using individuals’ unique SLaM hospital numbers. All identifiers were removed from the dataset before it was provided to the researchers. Individuals who have never had contact with SLaM services do not have a SLaM hospital number and so could not be matched. The anonymity requirements for HES data prevent linkage between HES and SHIELD data so individuals identified solely through HES inpatient data also could not be matched.

To allow matching on date of attendance we only counted the first attendance by an individual on a given date. Where attendances occurred close to midnight it was possible for the same attendance to appear in the two datasets on different days. To allow for this, dates were allowed to match with one day variance.

A sample of the first 50 attendances included in the SHIELD dataset but not contained in the EHR dataset were examined in detail. All clinical records in CRIS from the week of the ED presentation were extracted and read to determine if the individual had any contact with psychiatric services regarding the self-harm and if so the reason they had been missed from the EHR dataset. A similar examination of attendances missed by SHIELD was not possible because this would have required anonymised records to be re-identified to allow the corresponding ED notes to be examined. This is not possible under the information governance requirements for use of CRIS.

Individuals contained in the EHR dataset were compared to those missed by the SHIELD dataset in terms of sex, age, ethnicity and marital status. Differences were tested using chi squared tests.

## Results

### EHR dataset

The dataset contained 10,688 presentations for self-harm by 7444 individuals between April 2009 and December 2011. The characteristics of the attendances and individuals are shown in Tables 1 and 2. In the sample coded by both coders, testing of inter-rater reliability found that the kappa statistic for inter-rater reliability in identifying the presence of self-harm was 0.85; that for identifying the type of self-harm was 0.87.

**Table 1:** Attendances with self-harm at all four EDs April 2009-December 2011 by sex and method

		Male		Female		Total	
Total		4064	(38.0 %)	6624	(62.0 %)	10,688	
Type of self-harm							
	Self-poisoning	2867	(70.5 %)	5182	(78.2 %)	8049	(75.3 %)
	Self-injury	909	(22.4 %)	1130	(17.1 %)	2039	(19.1 %)
	Mixed^a^	97	(2.4 %)	176	(2.7 %)	273	(2.6 %)
	Other^b^	190	(4.7 %)	134	(2.0 %)	324	(3.0 %)
	Unknown	1	(0.0 %)	2	(0.0 %)	3	(0.0 %)

^a^Both self-poisoning and self-injury

^b^Includes attempted hanging, jumping from a height and immersion in water with intent to drown

**Table 2:** Individuals presenting with self-harm at all four EDs April 2009-December 2011 by age and sex

		Male		Female		Total
Total		2924	(39.3 %)	4520	(60.7 %)	7444
Age						
	<20	257	(8.8 %)	1067	(23.6 %)	1324
	20-24	428	(14.6 %)	846	(18.7 %)	1274
	25-29	422	(14.4 %)	592	(13.1 %)	1014
	30-34	365	(12.5 %)	434	(9.6 %)	799
	35-39	371	(12.7 %)	384	(8.5 %)	755
	40-44	362	(12.4 %)	409	(9.0 %)	771
	45-49	278	(9.5 %)	311	(6.9 %)	589
	50-54	170	(5.8 %)	197	(4.4 %)	367
	55+	270	(9.2 %)	280	(6.2 %)	550
	Unknown	1		0		1

### Validation of dataset

#### Matching datasets

SHIELD contained 2026 presentations by 1521 individuals in 2011 at St Thomas’ Hospital or King’s College Hospital. Of these, 79 individuals, accounting for 80 attendances (3.9 %) were missing SLaM hospital numbers and so could not be matched with EHR data. The EHR dataset contained 1998 attendances by 1493 individuals in the same period at these two hospitals. Fifty-one attendances (2.6 %) by 51 individuals were only identified through HES inpatient data and had never had any contact with SLaM services and hence did not have a SLaM hospital number and were not included in the matching process. It is likely, although not possible to test, that some of the data excluded from both datasets refer to the same attendances and individuals.

Fifteen attendances in the EHR dataset and 21 attendances in the SHIELD dataset were excluded because they were the individual’s second attendance that day. Fifty-five attendances were matched by allowing matches plus or minus one day, however there were 19 cases where this resulted in an attendance in the EHR dataset matching two attendances in SHIELD effectively excluding 19 attendances from the SHIELD dataset. Overall 121 attendances (5.9 %) and 71 individuals (4.7 %) in SHIELD and 66 attendances (3.3 %) and 51 individuals (3.4 %) in the EHR dataset were excluded from the validation exercise.

#### Proportion of cases detected

Between 1/1/2011 and 31/12/2011 in St Thomas’ and King’s College Hospital, there were 2503 attendances occurring in at least one of the EHR dataset and SHIELD. The number of attendances occurring in at least one dataset is treated here as the total number of possible attendances. Table 3 shows that both the SHIELD and the EHR datasets detected about three-quarters of total known attendances.

**Table 3:** Attendances and individuals presenting following self-harm at St Thomas’ Hospital and King’s College Hospital in 2011 identified by the EHR dataset and SHIELD datasets

	Attendances		Individuals	
EHR	1932	(77 %)	1442	(82 %)
SHIELD	1906	(76 %)	1450	(82 %)
Total	2503		1768

**Table 4:** Characteristics of individuals presenting following self-harm at St Thomas’ Hospital and King’s College Hospital in 2011 identified or missed by EHR dataset^a^

		EHR		Missing	
Total		1442		326	
Sex					
	*Missing*	*1*	*(0.07 %)*	*8*	*(2.5 %)*
	Female	847	(59 %)	171	(54 %)
	Male	594	(41 %)	147	(46 %)
	Total	1441		318	
Age					
	*Missing*	*1*	*(0.07 %)*	*8*	*(2.5 %)*
	<20	225	(16 %)	63	(20 %)
	20-24	279	(19 %)	44	(14 %)
	25-29	177	(12 %)	31	(10 %)
	30-34	170	(12 %)	41	(13 %)
	35-39	142	(10 %)	37	(12 %)
	40-44	159	(11 %)	32	(10 %)
	45-54	183	(13 %)	46	(14 %)
	55+	106	(7 %)	24	(8 %)
	Total	1441		318	
Ethnicity					
	*Missing*	*43*	*(3.0 %)*	*27*	*(8.3 %)*
	White	939	(67 %)	217	(73 %)
	Black	253	(18 %)	45	(15 %)
	Other	207	(15 %)	37	(12 %)
	Total	1399		299	
Marital status					
	*Missing*	*35*	*(2.4 %)*	*27*	*(8.3 %)*
	Divorced/separated	126	(9 %)	25	(8 %)
	Married/cohabiting	215	(15 %)	43	(14 %)
	Single	1,043	(74 %)	225	(75 %)
	Widowed	23	(2 %)	6	(2 %)
	Total	1,407		299	

^a^Comparing available data, no significant difference was found between those individuals identified and those missed for sex (*p* = 0.10), age (*p* = 0.16), ethnicity (*p* = 0.19) or marital status (*p* = 0.93)

There were 1768 individuals in at least one of the datasets. The number of individuals occurring in at least one dataset is treated here as the total number of possible individuals. Table 3 shows that again, the performance of both datasets is very similar, with each detecting 82 % of individuals.

The effect of the exclusions that occurred in the matching process was tested by repeating the validation exercise including all excluded individuals with the assumption that they were independent. There was very little difference in the proportions of attendances and individuals detected (results not shown here).

#### Attendances missed by the EHR dataset

There were 597 attendances that occurred in the SHIELD dataset but do not appear in the EHR dataset. Examination of a sample of 50 identified the reasons that attendances had been missed falling into three groups:

1. No record of attendance to find in CRIS or HES APC. In 26 cases the individual had not had any contact with psychiatric liaison teams within the general hospital and had not been admitted so did not appear in HES inpatient data. Of these, 16 had been transferred from another psychiatric setting (for example inpatient wards) to EDs for medical treatment for self-harm and were returned to that setting for psychiatric assessment. The remaining 10 did not receive any psychiatric assessment.
2. Record of attendance not extracted by our query. Thirteen cases were missed because incomplete or incorrect data or the query itself meant that they were not identified as ED attendances and/or the cause was not identified as self-harm.
3. Differences in the way extracted records were coded

In 11 cases, records were identified for coding by our query but not coded as self-harm due to differences in the definition of self-harm between the EHR dataset and SHIELD or miscoding. In one case the record had not been flagged for coding because if did not contain any of the self-harm keywords searched for by our query. Five were found to have been incorrectly coded as not self-harm when the extracted clinical records were reviewed again and so should have been included in the EHR dataset. Five were correctly coded as not a self-harm presentation according to our coding rules. In one case the individual had already previously presented to an ED for treatment following the incident described and so was excluded from the EHR dataset. In the other four cases no act of self-harm had occurred suggesting they were incorrectly included in the SHIELD dataset.

As noted above, information governance requirements precluded an examination of the reasons for cases present in the EHR dataset being missed in the SHIELD dataset.

#### Differences between the EHR dataset and missing data

The demographic characteristics for the 1442 individuals contained in the EHR dataset were compared with the 326 who were missed by this dataset and only present in SHIELD (data shown in Table 4). Demographic data were more likely to be unavailable for individuals missed from the EHR dataset. When the available data were compared we found no significant differences in sex, age, ethnicity or marital status distribution (p > 0.1 for all comparisons).

## Discussion

Reducing the numbers of presentations to EDs and improving the quality of their assessment has been identified as a national priority in the UK [14]. However, there is currently no adequate source of data to monitor the numbers of presentations to EDs in the England outside of a few study centres. We examined whether such a dataset could be produced from routinely available clinical data and Hospital Episode Statistics by querying EHRs and coding selected entries.

The dataset we produced detected 10,688 attendances to EDs, more than double the 4491 admissions following self-harm to the four hospitals studied for the same period. This reinforces findings from research in other areas of the UK [1] that the use of admission data alone for self-harm results in many cases being missed. Data for ED presentations cannot be considered to represent all self-harm occurring in an area, as it is known that many individuals who self-harm, especially those who self-injure, do not seek medical treatment [15]. However, those individuals presenting to EDs are likely to represent the more severe end of the spectrum of self-harming behaviour, particularly self-poisoning. Our dataset found that the majority of self-harm presenting to EDs was self-poisoning (75.3 %), a similar proportion to that seen in data from other UK EDs [10].

The use of routinely collected clinical data in EHRs allowed a dataset covering a large population and multiple EDs over several years to be assembled quickly and at low cost. However, the use of such data introduces important limitations. Despite all four EDs having policies of referring all individuals who present following self-harm to liaison psychiatry services it is likely that some individuals who attend EDs are not brought to the attention of the psychiatric liaison teams and so would be missed by the dataset. While all the liaison teams have policies of recording all referrals, even if the individual referred leaves the department before being seen, it is again likely that some individuals were missed in this case. Further, in order to extract the relevant data from the EHR we relied on the original recording of the data being accurate and complete.

The validation exercise allowed us to examine the effects of these limitations on our dataset. The SHIELD dataset we validated against was compiled by database scientists using audit forms and manual searches of ED notes on an on-going basis within the ED, a similar methodology to that used in other research on ED presentations following self-harm. Despite the different methodologies the performance of the two datasets was similar with both identifying about three quarters of all known attendances and over 80 % of individuals who attended at some point during the year. This suggests that, while the dataset we produced using the EHR method cannot be considered to contain all attendances with self-harm, it provides as good an approximation as other potential methods of collecting the same data at considerably reduced cost. It should be noted that we did not have access to free-text or semi-structured data items within the EDs' hospital information systems that could potentially have improved our ability to identify individuals who had self-harmed but who were never seen by psychiatric services.

Our further comparison of the individuals in the EHR dataset with those missed by the SHIELD dataset suggests that the limitations of the data were not leading to systematic bias in terms of sex, age, ethnicity or marital status. However, the detailed examination of the clinical records of a sample of those missed suggests that our dataset is more likely to miss presentations by individuals who are current psychiatric inpatients. This is probably because these individuals return to the psychiatric setting they came from to have a psychiatric assessment following assessment and treatment of their physical health needs and hence do not have contact with psychiatric liaison teams within the ED.

### Limitations of validation exercise

The validation exercise was constrained by the nature of the data available, the information governance rules for how they could be linked and requirements to maintain anonymity. In particular this prevented us from examining the reasons why SHIELD missed cases in the way we examined cases missed by the EHR dataset.

Individuals were matched using SLaM hospital numbers. All individuals entered into the SHIELD database are checked against the SLaM EHR by the database scientists and their SLaM hospital number added if they had one, so an individual did not have to have had contact with psychiatric services during the presentation in question in order to have a SLaM number in their SHIELD record. Nonetheless, a small proportion of the individuals in SHIELD have never had contact with any SLaM service and so did not have a psychiatric hospital number to be matched on. Additionally, the individuals in the EHR dataset solely identified through HES data do not have any identifiers they could be matched on due to the anonymity requirements for HES data and so could not be matched. The individuals lacking an identifier to match on in the two datasets may well be the same people, although this is impossible to test. For this reason we excluded these individuals from our main results comparing the two datasets. The amount of data excluded from each dataset due to this was small (3.9 % of attendances in SHIELD and 2.6 % of those in the EHR dataset). Sensitivity analysis checking the effect of the exclusions indicated they did not affect the results of the validation exercise.

In order to capture as many presentations as possible in the EHR dataset they were identified through a variety of ways; having a record of a referral to a psychiatric liaison team or featuring in HES ED attendance data or in HES inpatient data with an admission route through EDs. HES inpatient data only provides a date of attendance while referrals to psychiatric services will occur some time after the attendance time recorded in ED records. Hence time of attendance, where available in the EHR dataset, will not always match that of the same attendance in the Shield dataset, which takes time of attendance from ED records. These limitations on the available data made it impossible to match on both date and time accurately. We decided to match on date alone and to also to allow matches plus or minus one day to ensure matching of presentations that occurred close to midnight which could appear on different dates in different datasets. This means that where an individual presented more than once in 24 h their second attendance was excluded, something that occurred 40 times in the Shield dataset and 15 times in the EHR dataset. Again, the amount of data excluded from each dataset was small (1.8 % of attendances in SHIELD and 0.8 % in the EHR dataset) and so this is unlikely to have significantly affected the results.

Finally, when comparing the two datasets' performance we have assumed that the total number of attendances for self-harm is the number appearing in at least one of the datasets. This may be an underestimate as we cannot know how many attendances were missed by both datasets.

### Future development of the dataset

Our examination of a sample of those missed in our dataset highlights where it could be improved. Half of those missed did not have a record of contact with psychiatric liaison. Ways of reducing the number of such cases might be identified by auditing current clinical practice in the study EDs against their policies of referring all individuals presenting with self-harm to psychiatric liaison teams and recording all such referrals even if the patient does not wait to be assessed. Alternatively, further linkage of the psychiatric EHR to ED hospital information systems might make it possible to identify individuals who were discharged from the ED following self-harm without being referred to a psychiatric team. The other half of the missed attendances were due to miscoded or incomplete data. This suggests that future improvements in the quality of data inputted into the EHRs and refining of the query used to create this dataset could further improve the detection of cases.

In the future there is the potential to develop a more useful, routinely updated dataset from this pilot work. This would require more regular and frequent updates of the current ad hoc static linkages of HES data to the CRIS database, which could reduce the lag time on producing the dataset to three months. Current work within the Biomedical Research Centre for Mental Health developing the use of natural language processing software for the coding of free-text data into structured datasets may also allow the coding process for this dataset to be fully automated, further reducing the cost and time required to produce it.

## Conclusions

It was possible to use routinely collected clinical and administrative data to create a dataset of attendances to EDs following self-harm. The dataset included more than twice as many attendances as hospital admission for self-harm data, which is currently the only routinely available data source. Validation against a dataset collected using methods modelled on those used in previous research on self-harm presentations to EDs demonstrated that our EHR dataset detected a similar proportion of all attendances and individuals, and that those missed did not differ in terms of age, sex, ethnicity or marital status from those detected. The increasing use of EHRs by mental health and acute trusts presents an opportunity to monitor rates of self-harm in order to inform service planning and public health policy.

## Abbreviations

ED: Emergency department
CRIS: Clinical records interactive search
HES: Hospital episode statistics
SLaM: South London and Maudsley NHS Foundation trust
EHR: Electronic health record
NHS: National health service
NICE: National Institute for health and care excellence
ICD-10: International classification of diseases version 10
NIHR: National Institute for health research
